# Supplementary material for: M. mazei glutamine synthetase and glutamine synthetase-GlnK1 structures reveal enzyme regulation by oligomer modulation
Source: Nat Commun. 2023 Nov 15;14:7375. doi: 10.1038/s41467-023-43243-w (PMC10651883; doi:10.1038/s41467-023-43243-w)
Supplement: Supplementary file 1 — Supplementary Information [file 41467_2023_43243_MOESM1_ESM.pdf]

Supplementary information:

M. mazei glutamine synthetase and glutamine synthetase-GlnK1 structures reveal enzyme regulation by oligomer modulation

Maria A. Schumacher*, Raul Salinas, Brady A. Travis, Rajiv Ranjan Singh and Nicholas Lent

Department of Biochemistry, 307 Research Dr., Box 3711, Duke University Medical Center, Durham, NC 27710, USA

*Corresponding author: Maria A. Schumacher;

E-mail: Maria.Schumacher@duke.edu

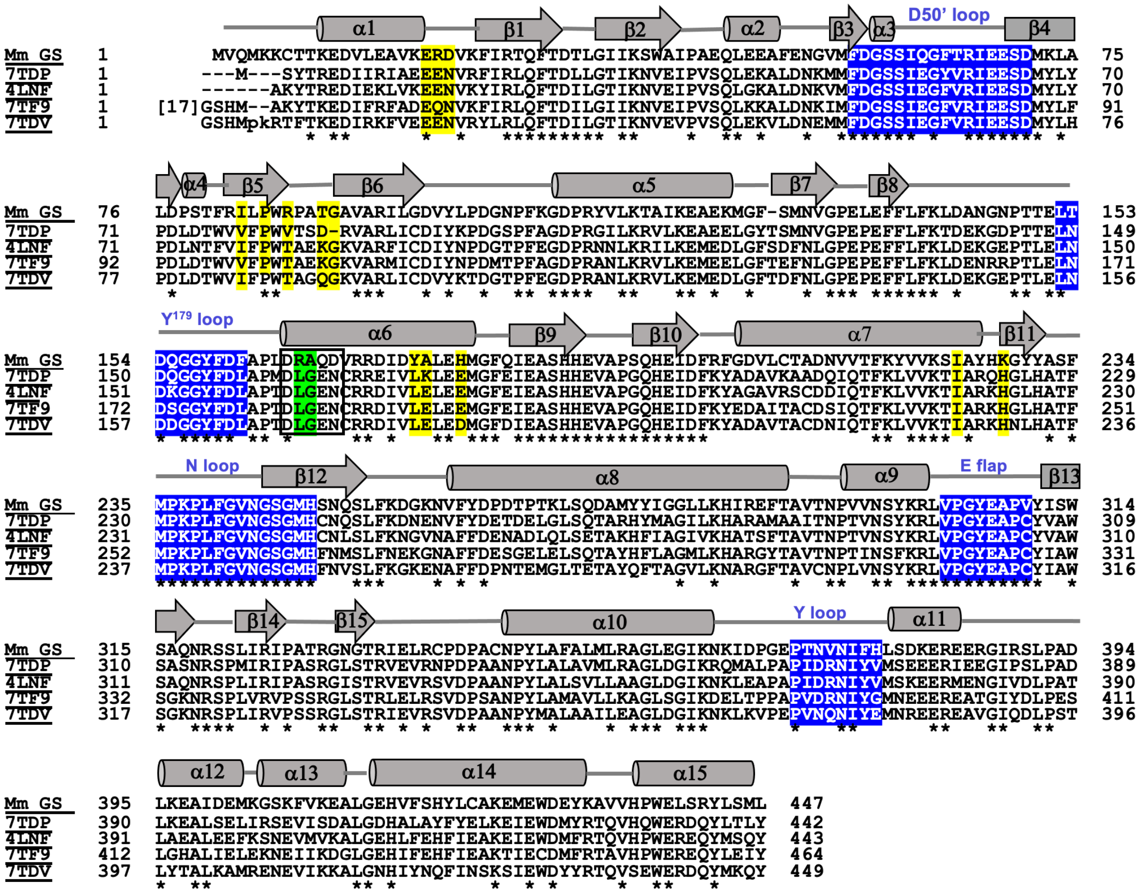

Supplementary Figure 1. Multiple sequence alignment of the *Mm* GS protein with GS proteins from Gram-positive bacteria. 7TDP, 4LNF, 7TF9 and 7TDV correspond to the structures for the *P. polymyxa* GS, *B. subtilis* GS, *L. monocytogenes* GS and *S. aureus* GS, which show 59.0%, 59.1%, 55.4% and 55.4% sequence identity with *Mm* GS, respectively. *Mm* GS active site loop regions are colored blue and labeled. *Mm* GS residues that contact GlnK1 (and their corresponding residues in the Gram-positive GS) are colored yellow. The region that shows structural differences in the hexamer interface when comparing *Mm* GS with the Gram-positive enzymes is boxed and residues corresponding to *Mm* GS residues Arg166 and Ala167 that were mutated to assess their roles in the interface and GS oligomer stability are highlighted in green. Asterisks under the sequence indicates conserved residues in all the GS.

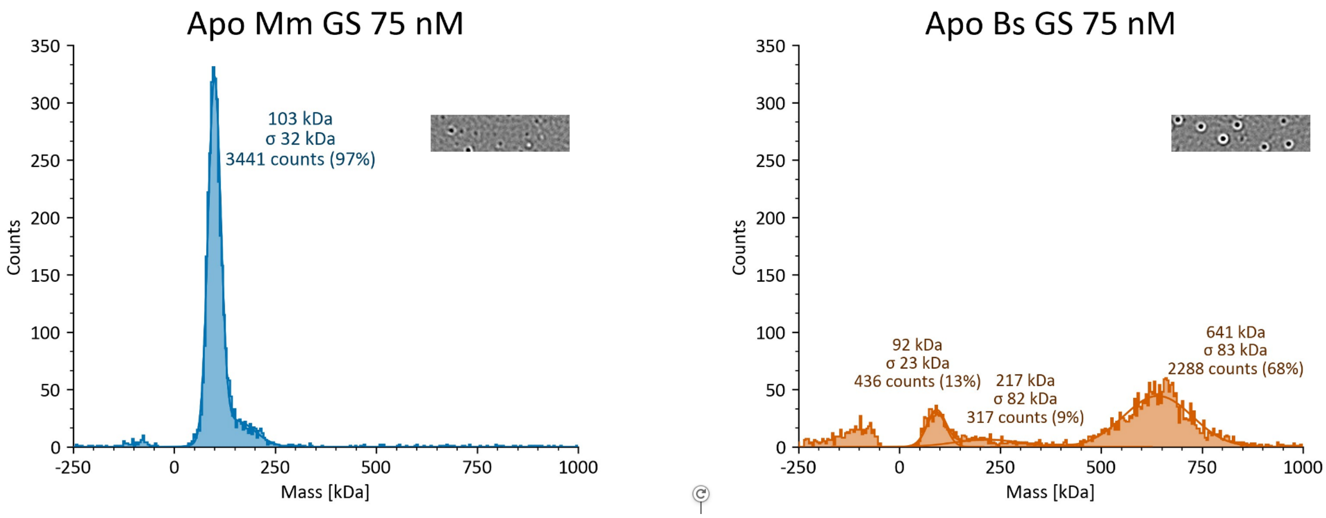

Supplementary Figure 2. Representative Mass Photometry (MP) of Apo *Mm* GS and Apo *Bs* GS at 75 nM concentration. Analyses of the samples revealed that only one band, corresponding to 103 kDa or a dimer, was present for the apo *Mm* GS, while three populations were found for the Apo *Bs* GS sample, at 92 kDa, 217 kDa, and 641 kDa corresponding to dimer, tetramer, and dodecamer, respectively. The samples were both in a buffer consisting of 20 mM Tris pH 7.5 and 150 mM NaCl**.** Inset images are single raw frame as representative of data acquisition video out of 6000 frames. For each sample, four technical repeats were performed.

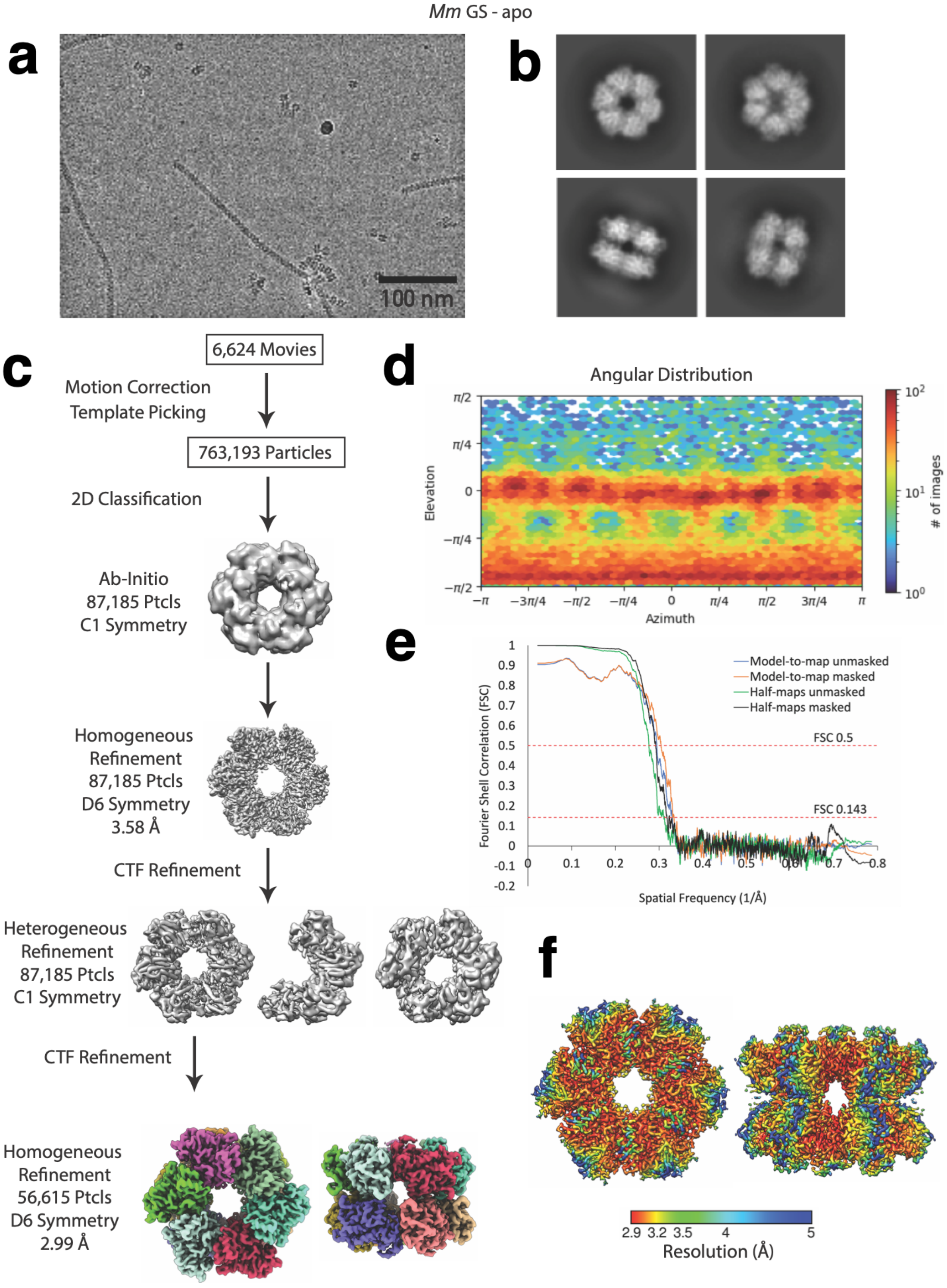

Supplementary Figure 3. Cryo-EM data processing and reconstruction of the *Mm* GS dodecamer. **(a)** A representative micrograph of *Mm* GS on a holey gold grid. (**b**) A subset of the 2D classes showing top and side views of the complex. (**c**) Summary of the data processing workflow. (**d**) Angular distribution plot of the final particle set. (**e**)Masked and unmasked half-map and model-to-map FSC curves. (**f**) Final sharpened map colored by local resolution.

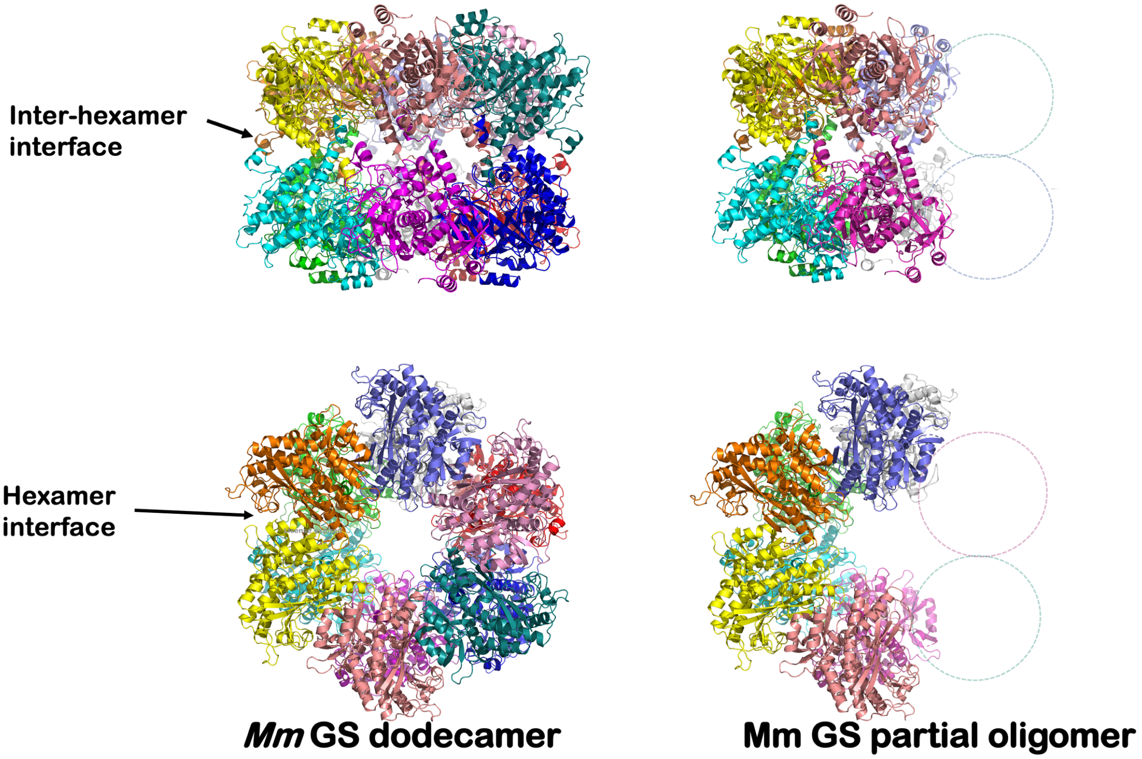

Supplementary Figure 4. Comparison of apo *mM* GS dodecamer and partial oligomer structures. Left, shows the *Mm* GS dodecamer organization with the subunits colored differently. Right shows the partial GS structure, with the subunits colored the same to highlight the missing subunits in the partial structure.

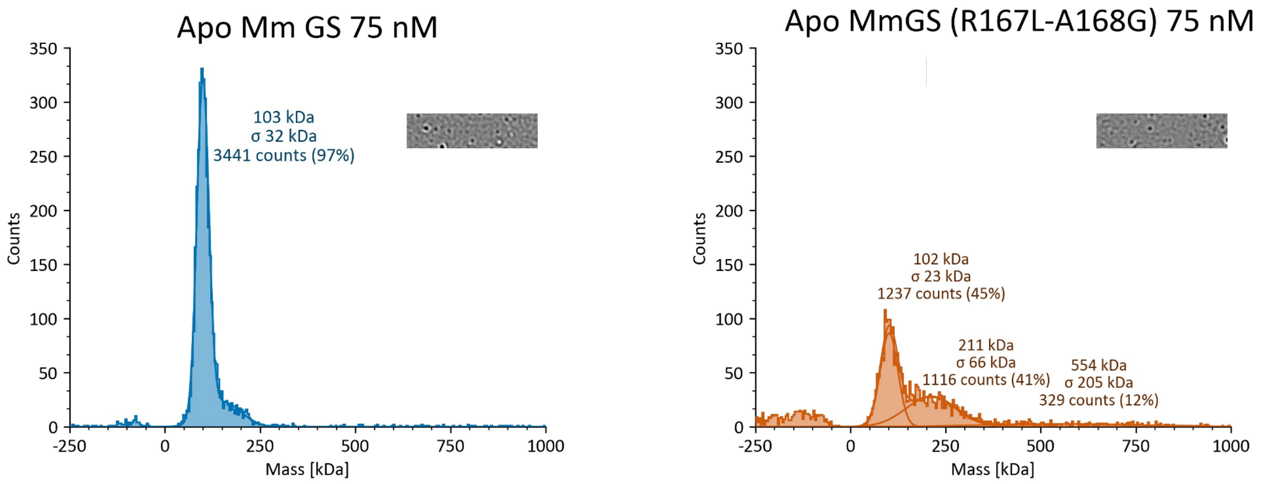

Supplementary Figure 5. Representative Mass Photometry of Apo *Mm* GS and Apo *Mm* GS (R167L-A168G) at 75 nM concentration. The apo Mm GS samples revealed one band at 103 kDa corresponding to dimer while three bands were observed for the Apo *Mm* GS (R167L-A168G) sample at 102 kDa, 211 kDa, and 554 kDa corresponding to dimer, tetramer/dimer of dimer, and dodecamer, respectively. Inset images are single raw frame as representative of data acquisition video out of 6000 frames. The buffer used for both samples was 20 mM Tris pH 7.5 and 150 mM NaCl. For each sample, four technical repeats were performed.

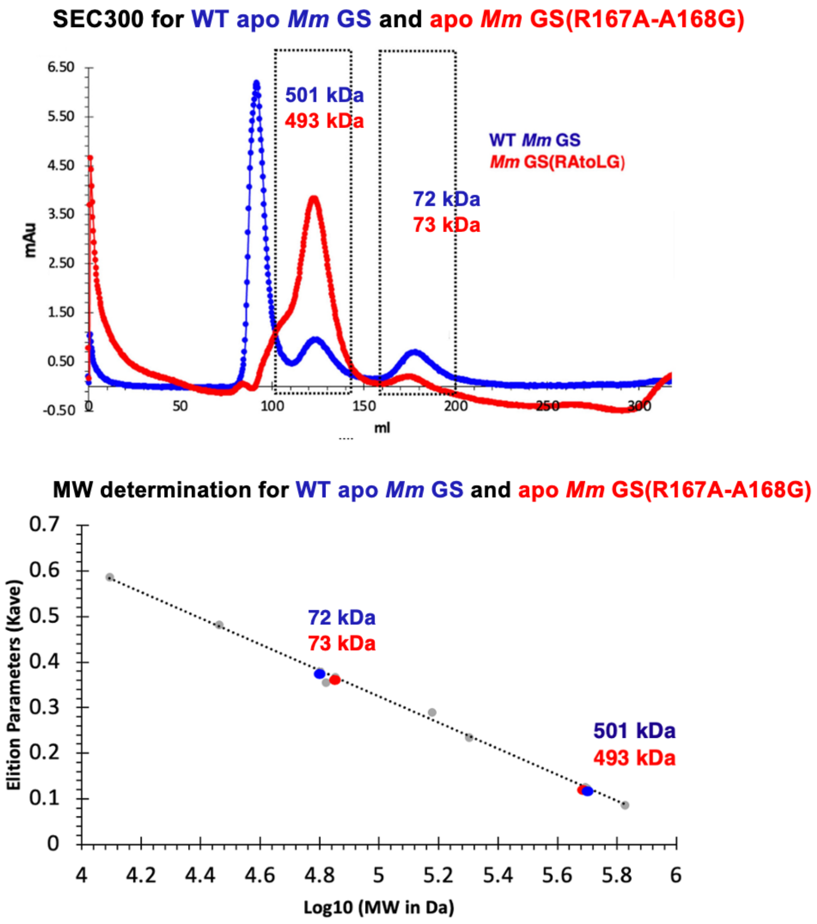

Supplementary Figure 6. SEC profiles of WT apo *Mm* GS (blue) and apo *Mm* GS(R167L-A168G) (red) proteins. Experiments were done at 1 mg/mL on Hi Prep 26/60_Sephacryl S-300 HR column. Top panel, profiles of the two GS proteins. The elution at ~125 ml for both proteins corresponds to a MW range of 350-534 kDa while the elution peaks for the proteins at ~180 correspond to a MW range of 56 to 88 kDa, consistent with a mixture of GS monomers and dimers. Lower panel, calibration curve done with six standards; Blue Dextran (2 mDa), Thyroglobulin (669 kDa), β-amylase (200 kDa), Alcohol dehydrogenase (150 kDa), Bovine Serum Albumin (66 kDa), Carbonic Anhydrase (29 kDa), and cytochrome C (12.4 kDa). The calibration curve was plotted with the y-axis Kave and the x-axis logarithm of the molecular weight (Log Mw). Kave = (Ve - Vo) / (Vc - Vo), where Ve = elution volume for the standard, Vo = column void volume = elution volume for Blue Dextran (94.6 mL), Vc = total column volume (320 mL). Straight line is the resultant calibration curve calculated from the data for molecular weight standards (R2 = 0.9971). The equation, Y = -0.2863X + 1.7555 from the calibration curve was used to calculate the experimental molecular weights. For the WT Mm GS, some of the protein aggregated and eluted at the void volume.

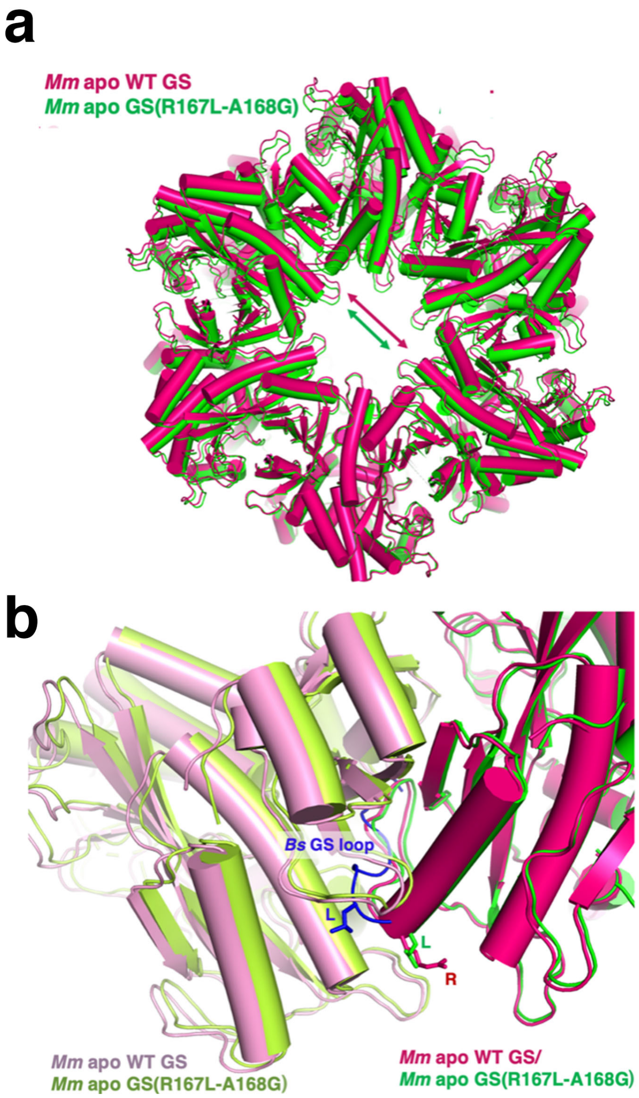

Supplementary Figure 7. Comparison of the apo *Mm* GS and apo *Mm* GS(R167L-A168G) structures. (a) In this comparison, one subunit was overlaid for each structure. Apo *Mm* GS is colored magenta and apo *Mm* GS(R167L-A168G) is green. Arrows in the center of the dodecamer highlight that the mutant dodecamer has a more compact dodecamer (with subunits more closely interacting). (**b**) Close-up of the region around residues 167 and 168 showing that the subunit shown with the 167-168 residues more closely interacting with the adjacent subunit. Also shown is the conformation of the 167-168 containing loop in the Bs GS structure, demonstrating that the corresponding region in the mutant does not adopt that conformation.

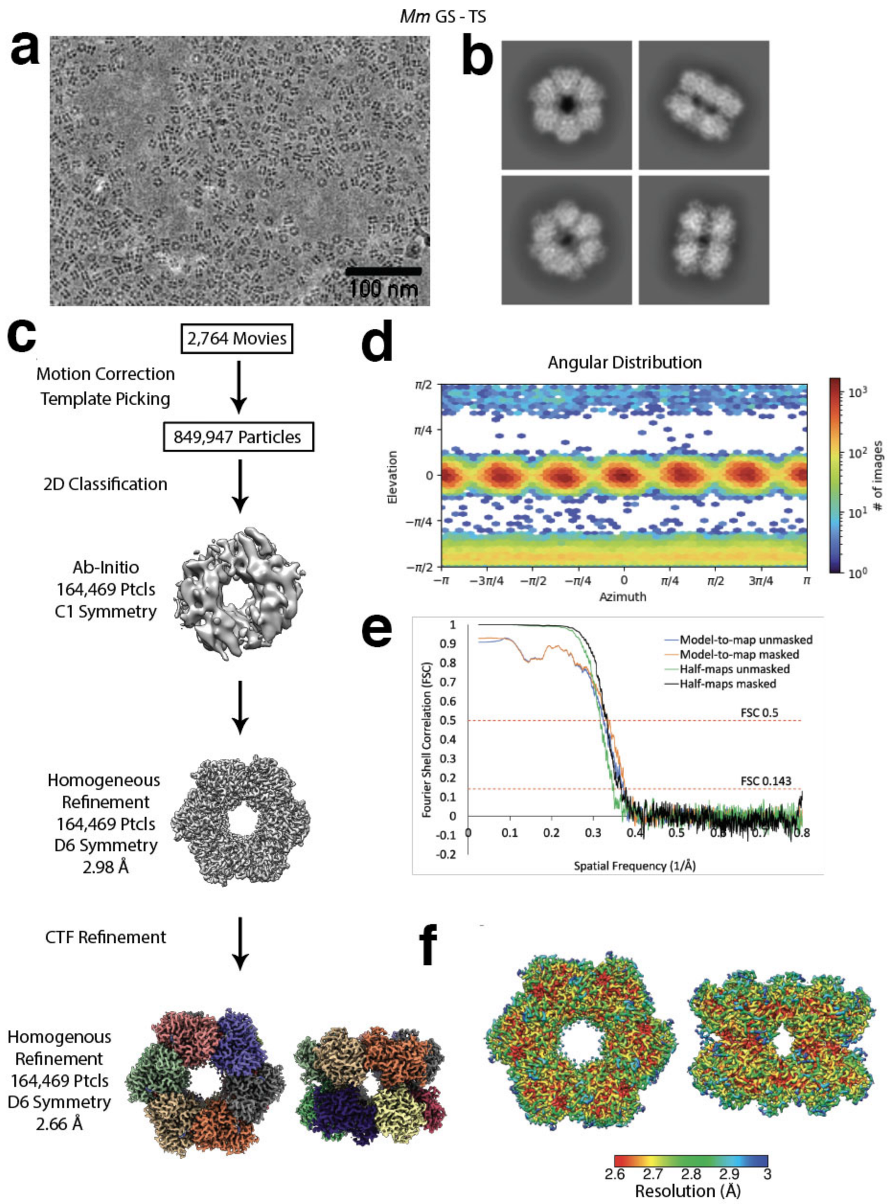

Supplementary Figure 8. Cryo-EM data processing and reconstruction of the *Mm* GS-transition state complex. (a) A representative micrograph of the *Mm* GS-TS complex on a holey gold grid. (**b)** A subset of the 2D classes showing top and side views of the complex. (**c)** Summary of the data processing workflow. (**d)** Angular distribution plot of the final particle set. (**e)** Masked and unmasked half-map and model-to-map FSC curves. (**f)** Final sharpened map colored by local resolution.

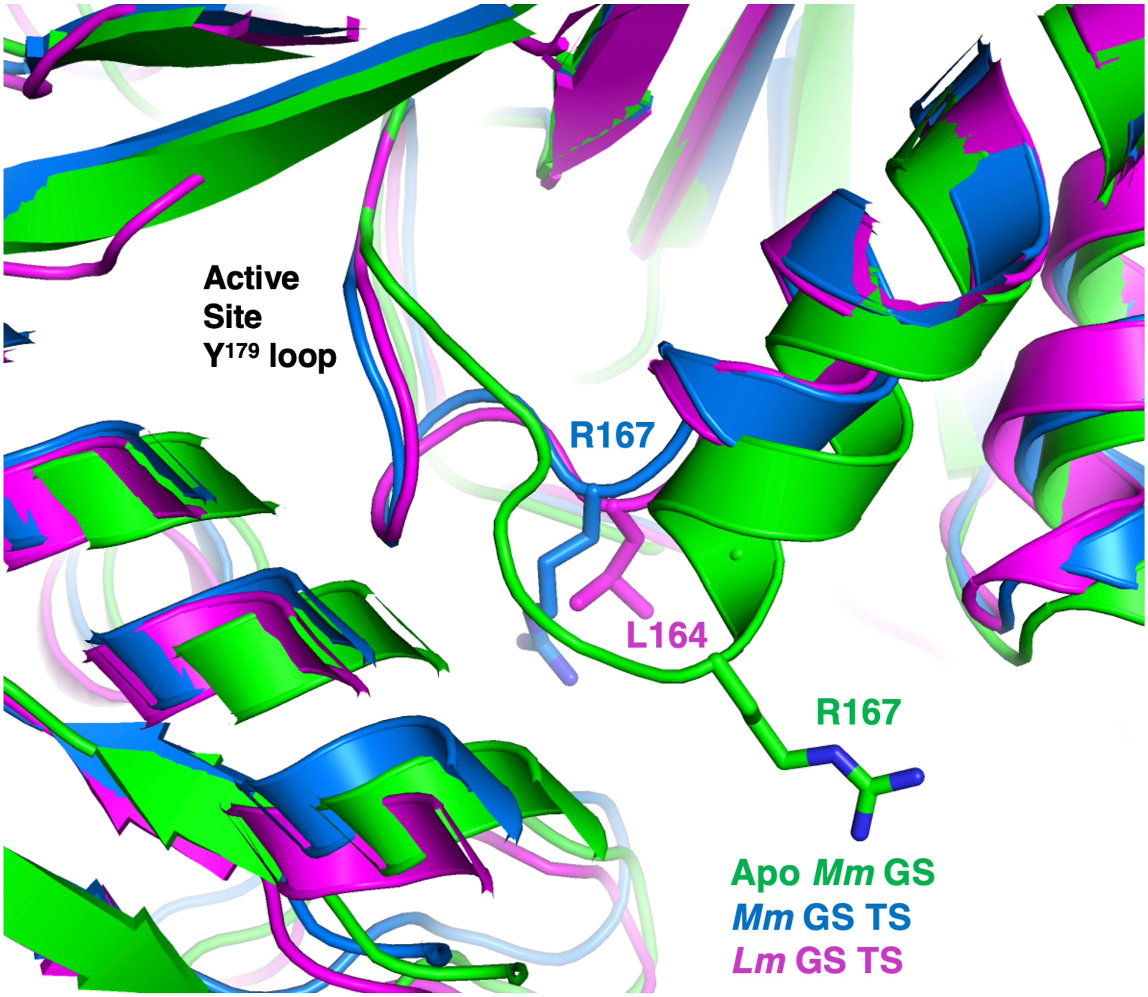

Supplementary Figure 9. Difference in *Mm* GS interface does not impact *Mm* GS TS formation. Shown is an overlay of the TS structures of apo *Mm* GS (green), *Mm* GS TS (blue) and *Lm* GS (magenta). The figure shows that the different conformation of the interface in *Mm* GS and the low G+C GS does not prevent *Mm* GS from forming the same TS structure.

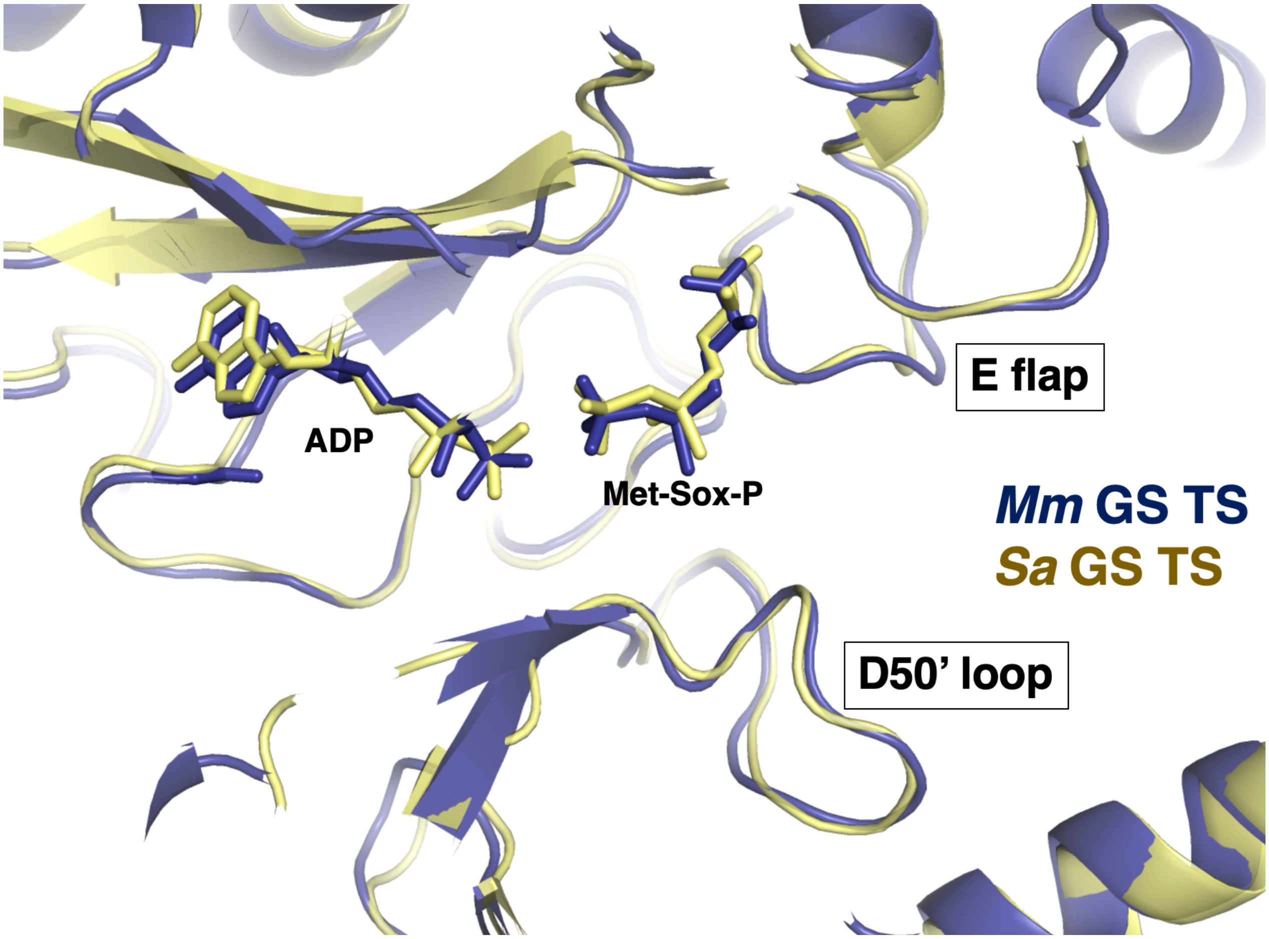

Supplementary Figure 10. *Mm* GS forms same overall TS as Gram-positive GS. Overlay of the TS structures of *Mm* GS and *Listeria monocytogenes* (*Lm*) GS (rmsd for 430 similar Cα atoms is 0.5 Å). The *Mm* GS TS structure is shown in dark blue and the *Lm* GS TS structure in dark yellow. Included are the positions of the ADP and transition state mimic, Met-Sox-P, formed during the reaction, showing they overlay. Also well superimposed are the GS structure. Critical active site loops, the D50' loop and the E flap, which adopt the same conformations in the structures, are labeled. Note, the *Sa, Bs* and *Pp* GS TS all adopt this same structure.

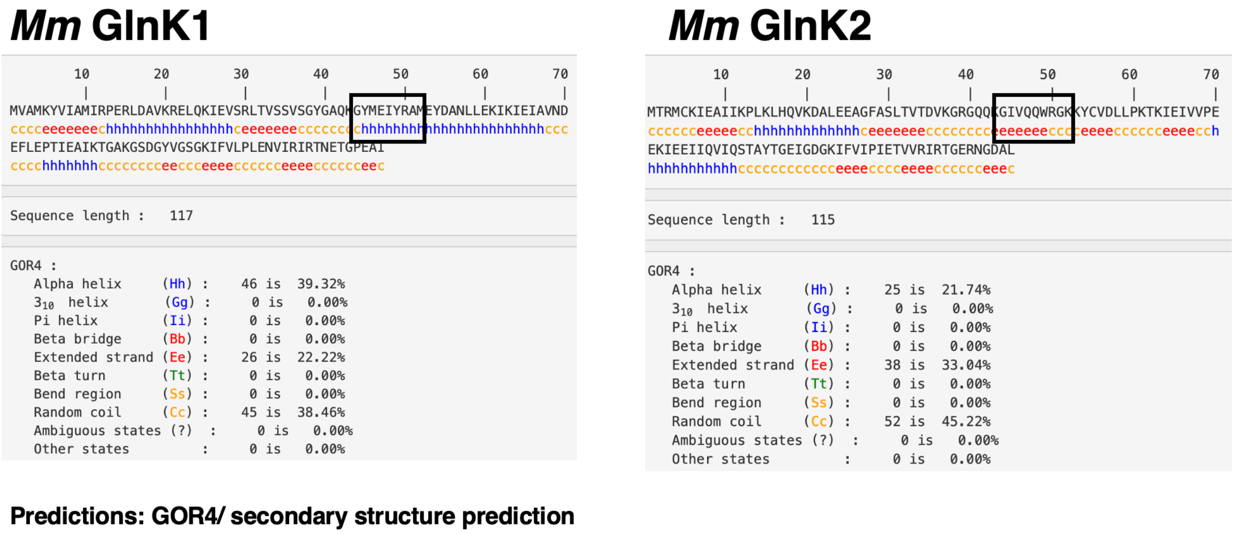

Supplementary Figure 11. Secondary structure prediction of *Mm* GlnK1 and *Mm* GlnK2. Residues predicted to lie within helices are indicated by blue 'h' while residues in strands are denoted in red 'e' and coiled ore disordered regions are shown as yellow 'c'. Boxed are the regions that form the GS interacting T-loop helix in the *Mm* GS-GlnK1 structure. Note that the showing that this region in GlnK1 is predicted to be capable of forming helix while the corresponding region in GlnK2 is predicted to prefer a strand secondary structural fold.

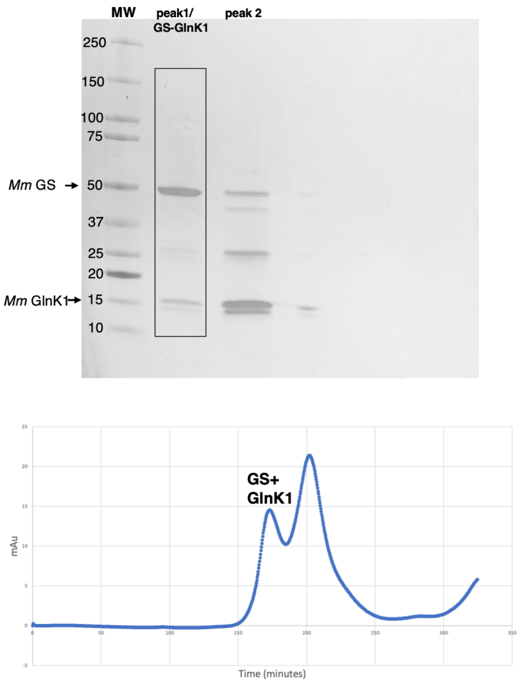

Supplementary Figure 12. SEC purification of *Mm* GS-GlnK1 complex for crystallization. The stoichiometry of *Mm* GS to GlnK1 was not known. Hence, a mixture was generated with excess GlnK1 (~2 fold excess over GS). The buffer for the mixture was 25 mM Tris pH 7.5, 150 mM NaCl, 0.1 mM βME, 2.5% glycerol and 1 mM $MgCl_2$. The first peak (boxed) contained the GS-GlnK1 complex, which was concentrated to 35 mg/mL and used in crystallization trials. The Molecular weight standard was Precission Plus Protein^{TM} Standard (BioRad). Cat # 161-0394. Below the gel picture is the resultant SEC profile with the peak containing the GS-GlnK1 complex labeled.

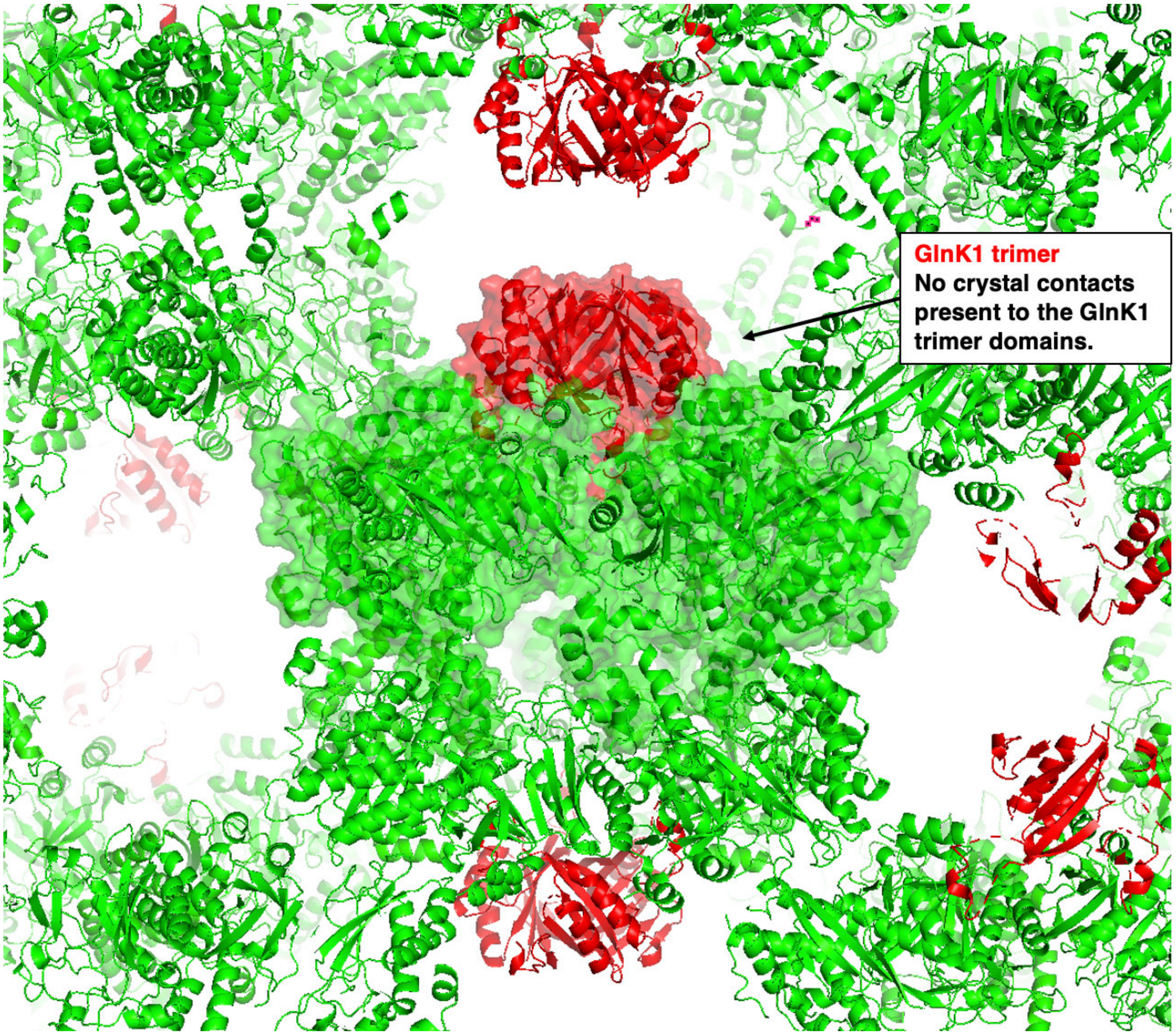

Supplementary Figure 13. Crystal packing in the *Mm* GS-GlnK1 crystal structure. The *Mm* GS subunits are colored green and the GlnK1 molecules are red. The crystallographic asymmetric unit (a GS hexamer and one GlnK1 trimer) is shown as a transparent surface. The biological unit is the *Mm* GS dodecamer bound to two GlnK1 trimers. The packing shows that the GlnK1 trimers are not involved in any crystal contacts, and just bound to their respective GS hexamer faces.

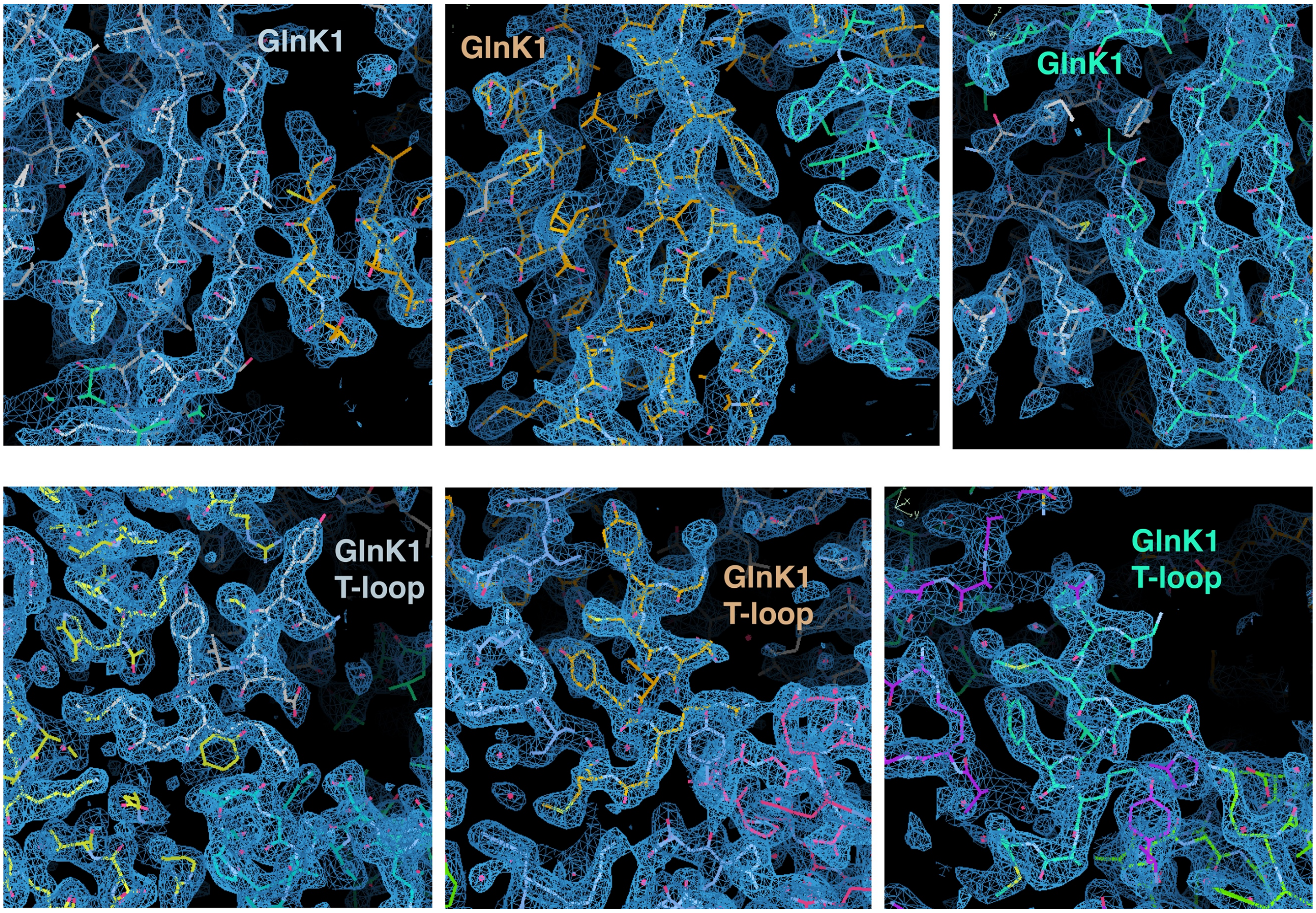

Supplementary Figure 14. **Representative regions of the 2*m*F_o-DF_c electron density map for GlnK1 in the *Mm* GS-GlnK1 complex.** Shown in the top panels are the SigmaA weighted electron density 2*m*F_o-DF_c map (contoured at 0.7 σ) of the trimer body of the GlnK1 showing density for the three subunits from the GS-GlnK1 structure (colored white, orange and light_green). Bottom panel shows the maps around the T-loop regions of each of the GlnK1 subunits bound to GS.

Supplementary Table 1. Cryo-EM data collection, refinement and validation statistics: *Mm* GS apo and *Mm* GS TS structures

	***Mm* GS8 - partial**	***Mm* GS12 - apo**	***Mm* GS-TS**
**Data collection and Processing**			
Electron microscope	Titan Krios (Duke)	Titan Krios (Duke)	Talos Arctica (UNC)
Pdb/EMD code	8TFC	8TFB	8TFK
Detector	K3	K3	K3
Magnification	22,500	22,500	54,900
Voltage (keV)	300	300	200
Electron exposure (e-/Å^2)	60	60	59
Defocus range (μm)	0.4-2.2	0.4-2.2	0.4-2.2
Pixel size (Å)	1.08	1.08	0.88
Total extracted particles (no.)	763,193	763,193	849,947
Refined particles (no.)	87,185	87,185	164,469
Movies	6,624	6,624	2,764
**Reconstruction**			
Final particles (no.)	11,802	56,615	164,469
Symmetry imposed.	C1	D6	D6
FSC 0.143 (unmasked/masked) (Å)	7.9/6.9	3.2/3.1	2.9/2.7
Map sharpening B-factor (Å^2)	0	119	109.9
**Refinement**			
**Model composition**			
Non-hydrogen atoms	27,640	42,108	82,557
Protein residues	3,488	5,316	5,280
Ions/Ligands	0	12	60
MolProbity score	1.24	1.20	1.92
Clash score	4.7	4.2	18.53
**Bonds (RMSD)**			
Bond lengths (Å)	0.004	0.003	0.006
Bond angles (°)	0.902	0.448	0.805
**Ramachandran analyses**			
Favored (%)	98.76	98.39	96.54
Allowed (%)	1.24	1.61	3.46
Disallowed (%)	0.00	0.00	0.00

Supplementary Table 2: Data collection and refinement statistics: *Mm* GS-GlnK1 complex and *Mm* GS(R167L-A168G)

	Mm GS-GlnK1	*Mm* apo GS(R167L-A168G)
**Data collection**		
Pdb code	8TGE	8UFJ
Space group	I2	I222
Cell dimensions		
a, *b*, *c* (Å)	138.0,178.0,169.0	137.3,167.6,176.9
α, β, γ (°)	90.0,90.4,90.0	90.0,90.0,90.0
Resolution (Å)	89.02-2.3 (2.36-2.30)*	45.52-2.45 (2.51-2.45)*
R_{sym} or R_{merge}	0.103 (0.618)	0.116 (0.908)
R_{pim}	0.068 (0.447)	0.075 (0.880)
$I/\sigma I$	8.3 (1.8)	11.1 (0.7)
Completeness (%)	99.6 (99.6)	90.4 (32.7)
Redundancy	3.1 (2.4)	5.7 (1.4)
CC(1/2)	0.994 (0.631)	0.999 (0.257)
**Refinement**		
Resolution (Å)	54.53-2.30	45.54-2.45
No. reflections	628555 (180243)	387075 (2274)
R_{work}/R_{free} (%)	16.5/21.8	19.0/22.1
B_{ave} (Å^2)		
GS Chain A	28.7	55.4
GS Chain B	30.2	54.6
GS Chain D	29.0	53.2
GS Chain Y	28.5	
GS Chain M	30.2	
GS Chain P	29.3	
GlnK1 Chain J/Tloop	75.8/48.6	
GlnK1 Chain Z/Tloop	79.9/46.5	
GlnK1 Chain G/Tloop	76.9/48.5	
Chain S/solvent	33.0	52.6
R.m.s. deviations		
Bond lengths(Å)	0.003	0.009
Bond angles (°)	0.623	0.978
**Ramachandran analyses**	97.6	97.5
Favored (%)	2.4	2.5
Disallowed (%)	0.0	0.0
Molprob. score	1.18	1.58

*Values in parentheses are for highest-resolution shell.
