# Supplementary material for: M. mazei glutamine synthetase and glutamine synthetase-GlnK1 structures reveal enzyme regulation by oligomer modulation
Source: Nat Commun. 2023 Nov 15;14:7375. doi: 10.1038/s41467-023-43243-w (PMC10651883; doi:10.1038/s41467-023-43243-w)
Supplement: Supplementary file 3 — Reporting Summary [file 41467_2023_43243_MOESM3_ESM.pdf]

nature portfolio

Corresponding author(s): Maria A. Schumacher

Last updated by author(s): Oct 17, 2023

Reporting Summary

Nature Portfolio wishes to improve the reproducibility of the work that we publish. This form provides structure for consistency and transparency in reporting. For further information on Nature Portfolio policies, see our Editorial Policies and the Editorial Policy Checklist.

Statistics

For all statistical analyses, confirm that the following items are present in the figure legend, table legend, main text, or Methods section.

n/a	Confirmed	
[ ]	[x]	The exact sample size (*n*) for each experimental group/condition, given as a discrete number and unit of measurement
[ ]	[x]	A statement on whether measurements were taken from distinct samples or whether the same sample was measured repeatedly
[ ]	[x]	The statistical test(s) used AND whether they are one- or two-sided<br>*Only common tests should be described solely by name; describe more complex techniques in the Methods section.*
[x]	[ ]	A description of all covariates tested
[x]	[ ]	A description of any assumptions or corrections, such as tests of normality and adjustment for multiple comparisons
[ ]	[x]	A full description of the statistical parameters including central tendency (e.g. means) or other basic estimates (e.g. regression coefficient) AND variation (e.g. standard deviation) or associated estimates of uncertainty (e.g. confidence intervals)
[ ]	[x]	For null hypothesis testing, the test statistic (e.g. *F*, *t*, *r*) with confidence intervals, effect sizes, degrees of freedom and *P* value noted<br>*Give P values as exact values whenever suitable.*
[x]	[ ]	For Bayesian analysis, information on the choice of priors and Markov chain Monte Carlo settings
[x]	[ ]	For hierarchical and complex designs, identification of the appropriate level for tests and full reporting of outcomes
[x]	[ ]	Estimates of effect sizes (e.g. Cohen's *d*, Pearson's *r*), indicating how they were calculated

Our web collection on statistics for biologists contains articles on many of the points above.

Software and code

Policy information about availability of computer code

Data collection	SerialEM 3.8.0, XDS (January 10, 2022), Refeyn AcquireMP v2023 R1 (version 2023)
Data analysis	cryoSPARC v3, Phenix 1.19, UCSF ChimeraX 1.5, Coot 0.9.6, Pymol 2.5.0, MolProbity 4.5.1, KaleidaGraph 4.5, serial # 8011073 (Synergy Software), Refeyn DiscoverMP v2023 R2 (version 2023).

For manuscripts utilizing custom algorithms or software that are central to the research but not yet described in published literature, software must be made available to editors and reviewers. We strongly encourage code deposition in a community repository (e.g. GitHub). See the Nature Portfolio guidelines for submitting code & software for further information.

Data

Policy information about availability of data

All manuscripts must include a data availability statement. This statement should provide the following information, where applicable:

- Accession codes, unique identifiers, or web links for publicly available datasets
- A description of any restrictions on data availability
- For clinical datasets or third party data, please ensure that the statement adheres to our policy

The structural data generated in this study by cryo-EM have been deposited in the Protein Data Bank under the codes 8TFC [http://doi.org/10.2210/pdb8TFC/pdb] and 8TFB [http://doi.org/10.2210/pdb8TFB/pdb] for the partial and dodecamer apo Mm GS structures and 8TFK [http://doi.org/10.2210/pdb8TFK/pdb] for the Mm GS-Met-Sox-P-ADP cryo-EM structure. The coordinates and structure factor amplitudes for the Mm GS-GlnK1 and the Mm GS(R167L-A168G) crystal structures have been deposited in the Protein Data Bank under the accession codes 8TGE [http://doi.org/10.2210/pdb8TGE/pdb] and 8UFJ [http://doi.org/10.2210/pdb8UFJ/pdb],

respectively. Other source data are provided as a source_data file.
The GS-GlnK1 structure was solved by molecular replacement (MR) using a hexamer of the Bs glutamine structure as a search model in Phenix (version 1.19) [PDB: 4LNN: https://www.doi.org/10.2210/pdb4LNN/pdb].
To model the Mm GS complexes, one GS subunit from the published S. aureus GS-glutamine-GlnR peptide structure was used and docked into each subunit density to generate oligomers (PDB: 7TF6 [http://doi.org/10.2210/pdb7TF6/pdb) was docked in the maps using UCSF Chimera X 1.5.

Research involving human participants, their data, or biological material

Policy information about studies with human participants or human data. See also policy information about sex, gender (identity/presentation), and sexual orientation and race, ethnicity and racism.

Reporting on sex and gender	N/A
Reporting on race, ethnicity, or other socially relevant groupings	N/A
Population characteristics	N/A
Recruitment	N/A
Ethics oversight	N/A

Note that full information on the approval of the study protocol must also be provided in the manuscript.

Field-specific reporting

Please select the one below that is the best fit for your research. If you are not sure, read the appropriate sections before making your selection.

☒ Life sciences ☐ Behavioural & social sciences ☐ Ecological, evolutionary & environmental sciences

For a reference copy of the document with all sections, see nature.com/documents/nr-reporting-summary-flat.pdf

Life sciences study design

All studies must disclose on these points even when the disclosure is negative.

Sample size	X-ray data were all collected to with 3-15 fold redundancy. For FP assays, sample size was determined to be adequate based on the consistency of measurable differences between groups, i.e. the variance that was noted between measurements for 3 technical samples. For FP, all experiments were done in triplicate. These sample sizes allowed for robust statistical analyses.<br>For MP, four measurements were taken for each individual sample (i.e particular GS or mutant). This sample size was evaluated to produce robust results based on a pilot study of the GS, which showed low variance/high reproducibility between technical replicates.<br>The structural data generated in this study have been deposited in the Protein Data Bank.
Data exclusions	No data were excluded from FP, MP experiments and the crystallographic data were processed with XDS using the default parameters. Cryo-EM micrographs were excluded based on the resolution of the CFT fit and if no particles were evident in the micrograph. Subsequently in data processing, particles in bad 2D and 3D classes (were artifacts such as ice) were excluded. No date points were excluded in FP, MP or SEC experiments.
Replication	Crystallographic data were also collected on one crystal per data set. Triplicates were performed for all FP assays and enzyme assays. MP experiments were done four times for each sample. All attempts at replication for FP, MP and SEC assays were successful. X-ray structural coordinates were tested and validated by the MolProbity server before deposition to the<br>Protein Data Bank and independent validation reports were then obtained from the Protein Data Bank upon data deposition. Three datasets for the GS-GlnK1 crystals were collected (and the one at the highest resolution was used for final analyses); two datasets were collected for the GS(R167L-A168G) and again, the highest resolution data set used in refinement. In both cases, the data sets replicated the same structure.
Randomization	Crystallography and cryo-EM samples were independent of each other. For assays other than MP (which were done 4 times) data were included in triplicate to ensure replication. Cryo-EM datasets were split into two for processing. Resolution was estimated by Fourier shell correlation between half-maps at the 0.143 threshold criteria, indicating resolution. For crystal structures,5% of the structure factor amplitude data were excluded from the refinement and used in the determination of Rfree.
Blinding	Researchers were not blinded during crystallographic or other data collection or analysis. Information regarding the samples is necessary to guide data collection and processing; For crystallographic analyses the identity of the sample (i.e. the specific GS state and its ligand) was necessary to enable model construction. For FP, enzyme assays and MP analyses the FP-ligand and sample (binder) identities were necessary to enable proper experimental setup (such as the needed concentration of protein) and measurement range. For SEC experiments the sample identity was essential to know so that the proper column could be utilized. For cryo-EM particle assignment to half sets as well as the corresponding resolution were performed automatically by the software.

Reporting for specific materials, systems and methods

We require information from authors about some types of materials, experimental systems and methods used in many studies. Here, indicate whether each material, system or method listed is relevant to your study. If you are not sure if a list item applies to your research, read the appropriate section before selecting a response.

Materials & experimental systems

n/a	Involved in the study
☒	☐ Antibodies
☒	☐ Eukaryotic cell lines
☒	☐ Palaeontology and archaeology
☒	☐ Animals and other organisms
☒	☐ Clinical data
☒	☐ Dual use research of concern
☒	☐ Plants

Methods

n/a	Involved in the study
☒	☐ ChIP-seq
☒	☐ Flow cytometry
☒	☐ MRI-based neuroimaging

Plants

Seed stocks	N/A
Novel plant genotypes	N/A
Authentication	N/A
